# Whole-Genome Sequencing of a Single Proband Together with Linkage Analysis Identifies a Mendelian Disease Gene

**DOI:** 10.1371/journal.pgen.1000991

**Published:** 2010-06-17

**Authors:** Nara L. M. Sobreira, Elizabeth T. Cirulli, Dimitrios Avramopoulos, Elizabeth Wohler, Gretchen L. Oswald, Eric L. Stevens, Dongliang Ge, Kevin V. Shianna, Jason P. Smith, Jessica M. Maia, Curtis E. Gumbs, Jonathan Pevsner, George Thomas, David Valle, Julie E. Hoover-Fong, David B. Goldstein

**Affiliations:** 1 McKusick-Nathans Institute of Genetic Medicine, Johns Hopkins University School of Medicine, Baltimore, Maryland, United States of America; 2 Predoctoral Training Program in Human Genetics, Johns Hopkins University School of Medicine, Baltimore, Maryland, United States of America; 3 Center for Human Genome Variation, Duke University School of Medicine, Durham, North Carolina, United States of America; 4 Department of Psychiatry, Johns Hopkins University School of Medicine, Baltimore, Maryland, United States of America; 5 Department of Cytogenetics, Kennedy Krieger Institute, Baltimore, Maryland, United States of America; 6 Department of Neuroscience, Johns Hopkins University School of Medicine, Baltimore, Maryland, United States of America; 7 Department of Neurology, Kennedy Krieger Institute, Baltimore, Maryland, United States of America; 8 Department of Pediatrics, Johns Hopkins University School of Medicine, Baltimore, Maryland, United States of America; 9 Greenberg Center for Skeletal Dysplasias, Johns Hopkins University School of Medicine, Baltimore, Maryland, United States of America; Stanford University School of Medicine, United States of America

## Abstract

Although more than 2,400 genes have been shown to contain variants that cause Mendelian disease, there are still several thousand such diseases yet to be molecularly defined. The ability of new whole-genome sequencing technologies to rapidly indentify most of the genetic variants in any given genome opens an exciting opportunity to identify these disease genes. Here we sequenced the whole genome of a single patient with the dominant Mendelian disease, metachondromatosis (OMIM 156250), and used partial linkage data from her small family to focus our search for the responsible variant. In the proband, we identified an 11 bp deletion in exon four of *PTPN11*, which alters frame, results in premature translation termination, and co-segregates with the phenotype. In a second metachondromatosis family, we confirmed our result by identifying a nonsense mutation in exon 4 of *PTPN11* that also co-segregates with the phenotype. Sequencing *PTPN11* exon 4 in 469 controls showed no such protein truncating variants, supporting the pathogenicity of these two mutations. This combination of a new technology and a classical genetic approach provides a powerful strategy to discover the genes responsible for unexplained Mendelian disorders.

## Author Summary

Metachondromatosis (MC) is an autosomal dominant condition characterized by exostoses (osteochondromas), commonly of the hands and feet, and enchondromas of long bone metaphyses and iliac crests. MC exostoses may regress or even resolve over time, and short stature is not characteristic of MC. Here, we sequenced the whole genome of a single patient with MC and used partial linkage data from her small family to focus our search for the responsible variant. In the proband, we identified an 11 bp deletion in exon four of *PTPN11*, which results in premature translation termination and co-segregates with the phenotype. In a second metachondromatosis family, we identified a nonsense mutation in exon 4 of *PTPN11* that also co-segregates with the phenotype. Germline gain-of-function missense mutations in *PTPN11* cause an overlapping but distinct group of dominant disorders with involvement of the face, heart, skeleton, skin, and brain, including Noonan syndrome (OMIM 163950), Noonan-like disorder with multiple giant cell lesion syndrome (OMIM 163955), and LEOPARD syndrome (OMIM 151100). Nonsense mutations in *PTPN11* have not been described in humans and the loss-of-function *PTPN11* mutations we report here are the first to be described in human disease.

## Introduction

Elucidation of the molecular bases of Mendelian disease has provided a rich resource for understanding genetic mechanisms, protein functions, the behavior of biological systems and mechanisms of disease [1]–[3]. Despite intense efforts with a variety of approaches, however, human geneticists have so far identified only ∼2,400 genes responsible for Mendelian phenotypes, or about 11% of the total number of protein coding genes in our genome. Currently, OMIM, a catalog of Mendelian disorders [4], lists >1,500 mapped Mendelian disorders for which the gene has yet to be identified, and practicing clinical geneticists know that there is an untold number of families with Mendelian disorders for which a molecular explanation or even clear mapping information has yet to be accomplished. Challenges that prevent harvesting this trove of biomedical information include the rarity of each disorder, small family sizes, reduced reproductive fitness of affected individuals, locus heterogeneity and diagnostic tools that query only a fraction of all biological systems [1]–[3], [5], [6].

The recent development of massively parallel DNA sequencing technologies has reduced the cost and increased the throughput of large-scale sequencing (LSS) and provides a new and potentially powerful way to identify virtually all of the mutations responsible for Mendelian disorders [7]. Indeed, at least two groups have used LSS coupled with hybridization strategies to “capture” the majority of known exons (the “exome”) for protein coding genes to identify genes responsible for three Mendelian disorders [6], [8], [9]. While there are many good reasons to use whole-exome sequencing (WES), including the lower cost (currently ∼5-fold) and the fact that exon variation is the most readily interpreted, it is also clear that WES will miss mutations of interest, including those variants that are either in exons that are not captured, are in non-exonic regulatory regions, or are structural variants. At least 1.4% of the disease variants listed in the Human Gene Mutation Database <http://www.hgmd.cf.ac.uk/ac/index.php> are in regulatory sequences and this is likely to be an underestimate given the traditional strategies used for mutation detection (largely PCR amplification and sequencing of exons). The same database lists about 7.5% of disease variants as structural variants. WES also requires higher average coverage levels than WGS, both because of the variable success of capturing different regions and because of “allelic imbalance” where one allele is preferentially captured over the other. For these reasons we elected to employ a WGS strategy.

Both WES and WGS identify a large number of sequence variants when compared to the reference sequence, making it important to prioritize variants. This can be based upon both the assessment of the likelihood of the variants being functional, especially in the WGS setting (Shianna et al. submitted), and on their frequency in healthy control populations. These approaches were used in the WES study that discovered the gene for Miller syndrome (OMIM 263750) by identifying functional mutations in the same gene in each of four unrelated patients and no controls (*6*). In addition to these approaches, it also is possible to utilize classical genetic strategies that depend on family structure and inheritance patterns to prioritize certain genomic regions. In our case, we show that it is possible to combine partial linkage information with other criteria for prioritizing variants to identify the genetic basis of a rare autosomal dominant disorder (metachondromatosis, OMIM 156250).

**Figure 1:**
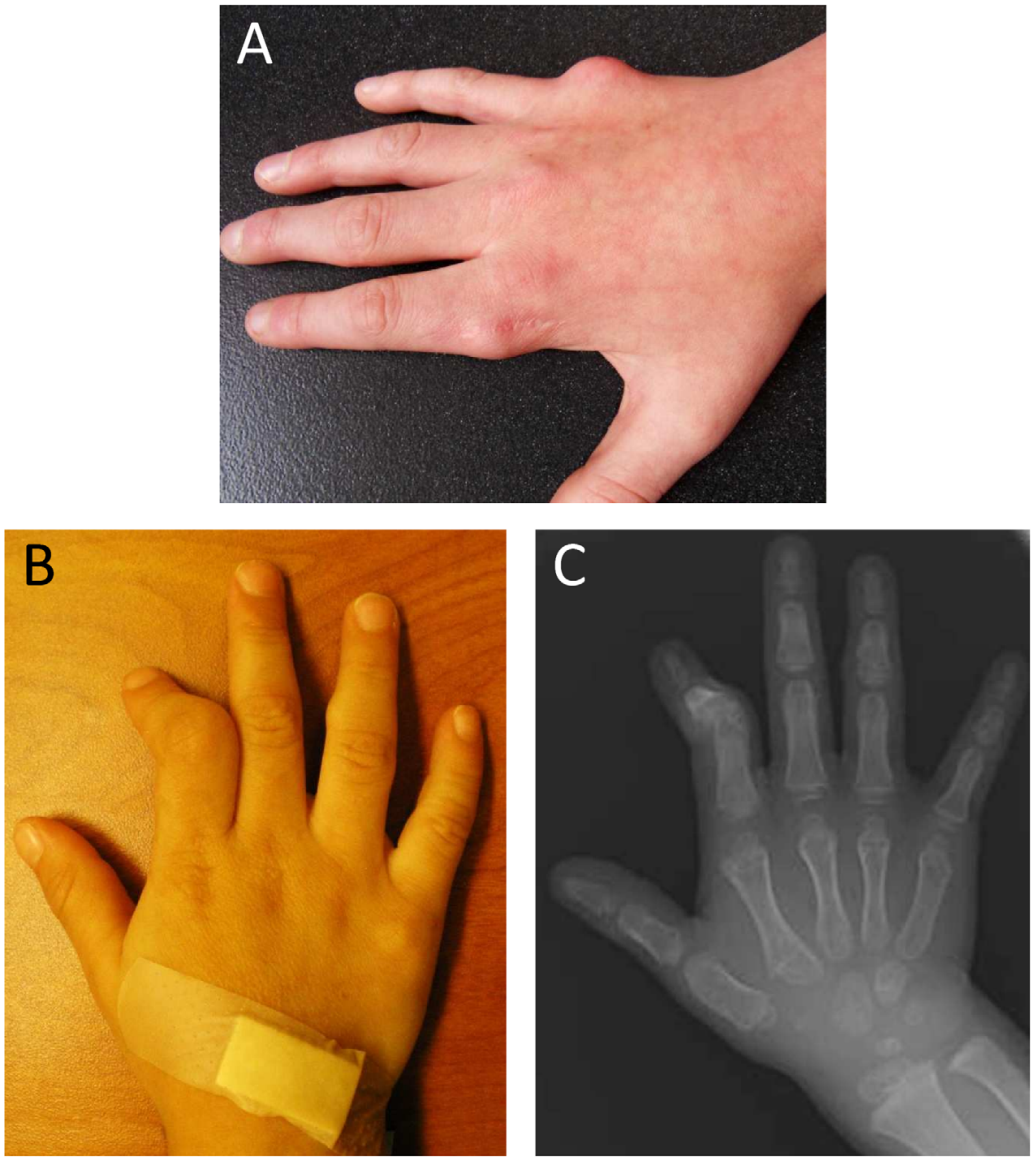
Manifestations of metachondromatosis. (A) Dorsal view of the right hand of individual III-2 in Pedigree 2 at age 12 years, with residual exostoses following partial surgical removal over the metacarpophalangeal (MCP) joint of the second digit and primary exostoses of MCP of the fifth digit. (B) Dorsal view of the right hand of individual V-1 in Pedigree 1 at three years, showing deformity of the second digit secondary to exostoses of the middle phalanx and an exostoses over the middle phalanx of the fourth digit. (C) Radiograph of the dorsal view of the right hand of individual V-1 in Pedigree 1 at three years.

The condition we have studied, metachondromatosis (MC, OMIM 156250), is an autosomal dominant condition characterized by exostoses, commonly of the bones of the hands and feet, and enchondromas of the metaphyses of long bones and iliac crest. It was first described by Maroteaux in 1971, based on clinical observation of the six affected individuals from two families [10]. Shortly thereafter, Lachman reported a young male with enlarging, painless, hard lumps on multiple fingers with concurrent long bone metaphyseal and iliac irregularities, both proven by histopathology to be classic exostoses and enchondroma, respectively [11]. The enchondromas often have a “striated” appearance in radiographs, and in MC both types of lesions typically appear in childhood and may regress or even resolve over several years [10], [11] (Figure 1). This phenomenon likely contributes to the incomplete penetrance that has been described in MC families [11], [12]. The exostoses of MC differ in location, orientation and duration from those observed in a related set of phenotypes known as the hereditary multiple exostoses syndromes (MES I and II, OMIM 133700 and 133701). The exostoses of MESs rarely resolve and can cause permanent deformity [11]–[13]. The osteochondromas of MESs typically point away from the adjacent epiphysis and rarely affect the hands or feet, while those of MC point toward the epiphyses and usually present on the hands and feet [11], [13]. Though palpable, the exostoses of MC may not be calcified and therefore may be radiolucent [14], in part depending on the timing of the clinical exam and radiography in the lifespan of a given lesion. The enchondromas of MC are similar to those of Ollier disease (OMIM 166000, also known as multiple enchondromatosis) but the latter disorder usually lacks exostoses. Mutations in *EXT1* and *EXT2*, located respectively at 8q24 and 11p11–p12, have been identified in 70% of MES cases [15].

## Results

### Linkage data

**Figure 2:**
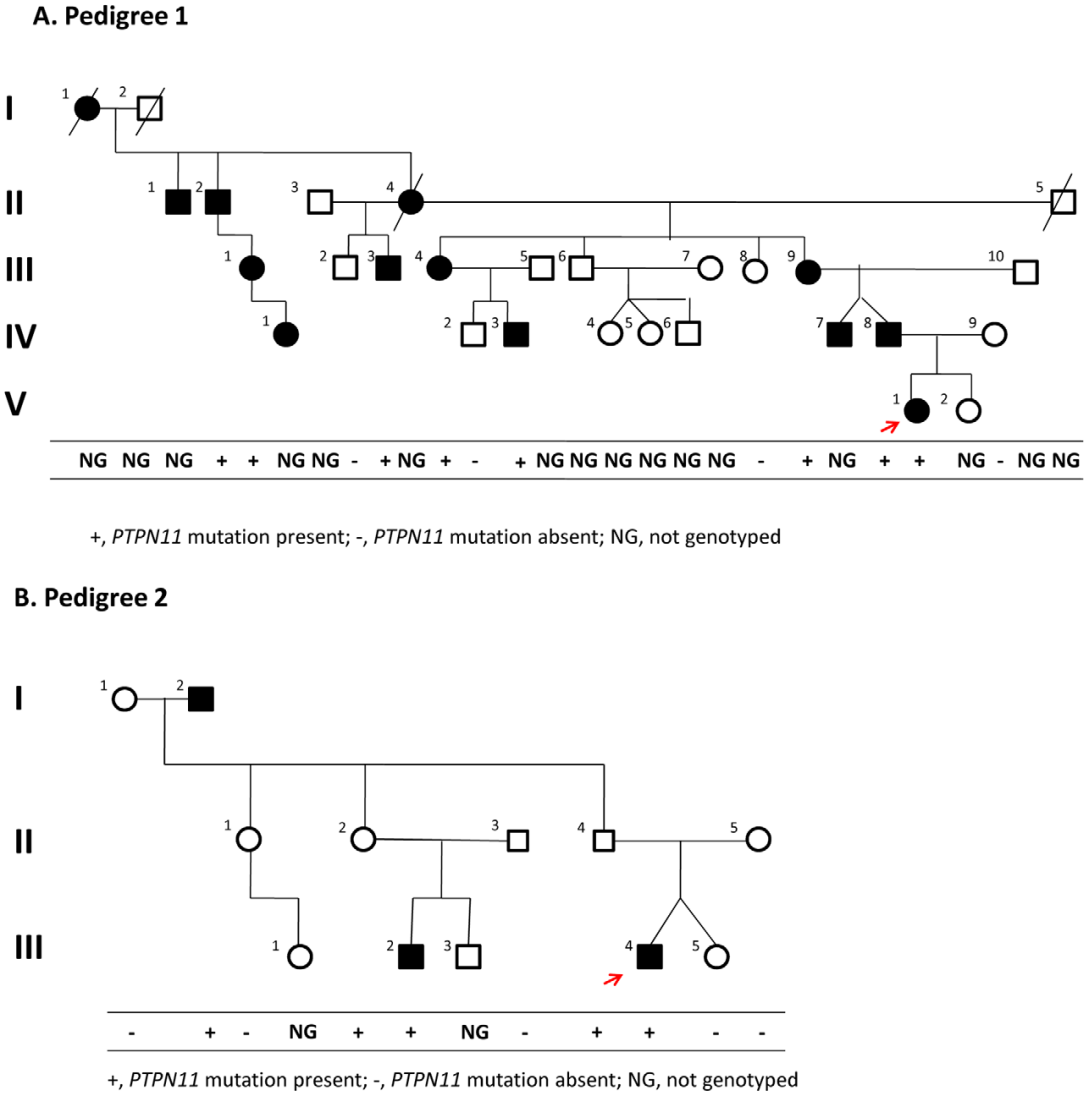
Pedigrees. Of families 1 (A) and 2 (B). The red arrow indicates the proband in each family. Segregation of the causal variant with the disease is shown in each family. Although III-1 and IV-1 in pedigree 1 were originally reported to be unaffected, we had the opportunity to examine them during the preparation of this manuscript and found that both had exostoses.

At the outset of our studies, we had access to a single MC family (Pedigree 1) and had not personally examined individual III-1, who was reported to be unaffected. We performed linkage analysis using multiple individuals from Pedigree 1 (Figure 2A) and found six regions with positive LOD scores. Three of these had LOD scores of 1.8 or higher: one at 7p14.1 (39.5–43.1 Mb) showed complete linkage and a LOD of 2.5 (the maximum possible in this family, resulting from perfect co-segregation); two more, 8q24.1 (129.3–141.2 Mb) and 12q23 (106.0–116.4 Mb), were each consistent with one non-penetrant individual and a LOD score of 1.8. Three other regions had LOD scores between 1.0 and 1.5; one was at 2p25 (3.9–10.0 Mb), one at 5q12.1 (60.6–62.5 Mb) and one at 9q31.1–q33.1 (111.1–119.3). We considered these six regions as showing suggestive evidence for the presence of a causal variant, and therefore concentrated attention on the approximately 42 Mb (767 Kb exonic) of included sequence.

### Evaluation of sequence data

We performed WGS on a single patient (V-1) from Pedigree 1, to an average of 31.8× coverage. In the 42 Mb of candidate regions defined by our linkage results, 95% of the exonic sequence was covered at a depth of >10×. We also sequenced eight unrelated controls and used data available from dbSNP. In the 7p14.1 (14 RefSeq genes), 8q24.1 region (27 RefSeq genes), 2p25 region (20 RefSeq genes), 5q12.1 region (7 RefSeq genes), and 9q31.1–q33.1 (71 RefSeq genes) regions we found no variants unique to the patient genome with a high likelihood of functional significance (stops gained and frameshifting indels). However, in the 12q23 region (105 RefSeq genes), we identified one frameshifting indel, an 11 bp deletion extending to but not including the 3′ base in exon 4 of *PTPN11* (c.514_524del11, see Supplementary Figure 4 in Text S1). This deletion shifts the reading frame, leading to a new sequence of 12 codons followed by a premature stop codon. We verified these results by direct Sanger sequencing of the *PTPN11* exon 4 amplicon.

### *PTPN11* mutations

We confirmed the segregation of the *PTPN11* deletion with the affected status in members of Pedigree 1 using a PCR assay (Supplementary Figure 3 in Text S1); all affected members carried the deletion. Additionally, one apparently unaffected individual (III-1), the same individual that was scored as non-penetrant in the linkage analysis, was also heterozygous for the *PTPN11* deletion. After obtaining these results, we had the opportunity to examine III-1 and found that she was affected with bilateral internal exostoses of her mandible and that her daughter (IV-1) was also affected with an exostoses of her right proximal tibia.

These WGS results together with the segregation analysis suggested that the *PTPN11* deletion was responsible for MC in Pedigree 1. To test this hypothesis, we identified a second family segregating MC as an autosomal dominant trait (Pedigree 2) (Figure 2B). We sequenced all *PTPN11* exons and flanking splice sites in individual III-4 of Pedigree 2 and found a heterozygous nonsense mutation, p.R138X, resulting from a C to T transition at position 111,375,382 in exon 4. This mutation abolishes the recognition site for *Rsa*I, and we used PCR amplification of *PTPN11* exon 4, followed by restriction of the amplicon with *Rsa*I, to follow the segregation of p.R138X in members of Pedigree 2. All affected individuals were p.R138X heterozygotes, as were two apparently unaffected individuals, II-2 and II-4, who were adults when examined. These results are consistent with the conclusion that this nonsense mutation is responsible for MC in affected individuals in pedigree 2, with non-penetrance in individuals II-2 and II-4, both of whom have affected children.

As a further test of the significance of the mutations we identified, we amplified and successfully Sanger sequenced *PTPN11* exon 4 in 469 control, unrelated individuals of whom 60% were of European descent similar to that of our two affected families, 11% were African-American, 11% were East Asian and 18% were of other ethnicities. We found no examples of either of these mutations, nor any other variants in exon 4 predicted to result in the loss of *PTPN11* function.

## Discussion

*PTPN11* encodes the protein tyrosine phosphatase SHP-2, which is an src homology-2 (SH2)-containing protein tyrosine phosphate (PTP) that is highly conserved among metazoans and plays a central role in RAS/MAPK signaling downstream of several receptor tyrosine kinases including EGFR and FGFR [16]. The N-terminal half of SHP-2 contains two SH2 domains (N-SH2 and C-SH2), while the C-terminal half contains the catalytic PTP domain. SHP-2 is ubiquitously expressed. Activation of SHP-2 has a positive effect on RAS/MAPK signal transduction in most contexts. Germline gain of function missense mutations in *PTPN11* cause an overlapping but distinct group of dominant disorders with involvement of the face, heart, skeleton, skin and brain, including Noonan syndrome (OMIM 163950), Noonan-like disorder with multiple giant cell lesion syndrome (OMIM 163955) and LEOPARD syndrome (OMIM 151100) [17], [18]. Nonsense mutations in *PTPN11* have not been described in humans, but in mice a gene-targeted mutant *Ptpn11* allele that deletes codons 46–110 is an early developmental recessive lethal [19], [20]. No phenotype was described in the heterozygotes, but the murine counterpart of MC could easily be overlooked and it would be useful to re-examine *Ptpn11+/−* mice [20]. The loss of function *PTPN11* mutations we report here (c.514_524del11 and p.R138X) are the first to be described in human disease. Not surprisingly, MC has essentially no phenotypic overlap with the other disorders caused by *PTPN11* mutations, with the possible exception that one affected individual in Pedigree 2, I-2, a 71-year-old male, has multiple truncal lentigenes (Supplementary Figure 1 in Text S1). How, or if, this is explained by a pathophysiologic overlap with the more extensive and earlier age of onset lentigenes characteristic of the LEOPARD syndrome is not clear.

Incomplete penetrance is a well-described feature of MC and many other dominant disorders and complicates co-segregation tests of candidate causative mutations. Interestingly, in Pedigree 1, individual III-1 was initially reported as unaffected and her status influenced our linkage analysis. However, during the preparation of this manuscript, we had our first opportunity to examine III-1. She has bilateral, internal exostoses of her mandible. Thus, her original classification as non-penetrant was incorrect.

Our results suggest that *PTPN11* and other members of the RAS/MAPK pathway should be examined in related and as yet unexplained Mendelian phenotypes such as Ollier's disease, Mafucci syndrome (OMIM 166000) and the trichorhinophalangeal syndrome type II (OMIM150230). Additionally, it is interesting to speculate on the focal nature and limited duration of the enchondromas and exostoses in MC. The local nature and childhood onset of these lesions suggests the possibility that a second mutational event is required for their appearance, a possibility that can be tested by examining *PTPN11* in these tumors. The reason for spontaneous regression of some of these lesions is unclear, but may be due to the developmental maturation of the affected tissue.

Our study adds to a small but growing list of examples where genome-wide sequencing approaches have successfully identified rare, high-penetrant risk factors for disease. Ours is one of the first to take the whole-genome approach, although the variant we identified would have been found using either WGS or WES. However, two key distinguishing features of this study are that discovery of the disease-causing variant resulted from the initial sequencing of only a single patient genome and that weak linkage evidence helped to identify regions most likely to harbor the causative variant. The linkage evidence present in this family restricted our search for the causal variant to 42 Mb, which contained only one protein-truncating variant unique to our sequenced case. Without this linkage evidence, sifting through the 109 protein-truncating variants unique to this genome (19 stops gained, 90 frameshifting indels) to find the causal variant would have been a difficult task. It would also have been difficult to sift through the many unique variants in the linked regions if it were not possible to assess the function of all identified variants, whether previously known or novel as the one we found. We also note that generating the necessary sequence data for all genes in the 42 Mb implicated region in the first family would be a daunting task using traditional sequencing approaches. This paradigm of WGS with the prioritization of variants by predicted functional consequence and frequency in controls combined with information gleaned by classical genetic approaches should prove effective for other unexplained Mendelian phenotypes and may also prove effective in the study of more common diseases that show modest linkage evidence.

## Materials and Methods

Informed consent was obtained through a Johns Hopkins Medical Institutions IRB approved protocol and a Duke University IRB approved protocol.

Individuals with MC from two families were identified and recruited from the Johns Hopkins Hospital Genetics Clinic. Informed consent was obtained through a Johns Hopkins Medical Institutions IRB approved protocol. Family 1 was a four-generation family comprised of 12 individuals for whom we had DNA. Seven individuals were classified as “affected” and five were “unaffected”. Family 2 was a three-generation family of three “affected” individuals and seven “unaffecteds” for whom we had DNA.

**Figure 3:**
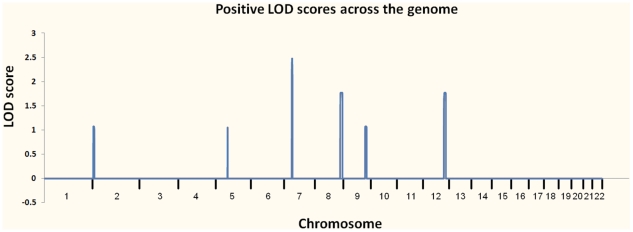
Plot of linkage results from Pedigree 1. The regions investigated for causal variants were 7p14.1, 8q24.1, 12q23, 2p25, 5q12.1, and 9q31.1–q33.1.

In preparation for interpreting the results of our WGS, we performed linkage analysis on Pedigree 1 (Figure 2A) by genotyping 7 family members using the Illumina HumanHap 550 Genotyping BeadChip v1.0 and 5 family members with Illumina Human 610-Quad v1.0 Genotyping BeadChip. We selected ∼2% of the genotyped SNPs (10,763 autosomal SNPs), requiring that they were heterozygous in at least 4 genotyped individuals, and used them for parametric linkage as calculated with the Merlin linkage analysis software [21] assuming a dominant model with reduced penetrance (set at 0.8). We also analyzed allele sharing and determined non-penetrant individuals using the tools pediSNP and SNPduo (Supplementary Figure 2 in Text S1). Figure 3 shows the results of the linkage analysis across all autosomes.

We performed WGS on a single individual (V-1) from MC pedigree 1 with an Illumina Genome Analyzer II. The sequence runs were paired-end 75 base pair reads, with 110 billion bases passing the Illumina analysis filter. To assess overall coverage, all gaps (stretches of N's) in the reference genome (Ensembl build 36 release 50) were excluded, resulting in the reference having 2,855,996,286 bases. After accounting for PCR duplicates and reads that did not align to the reference genome, our genomic coverage was 31.8×. We defined a “covered” base as one with at least five reads with a Phred-like consensus score greater than zero. Using these criteria, we covered approximately 97.8% of the reference genome. We then used data from eight control genomes that we had sequenced to an average coverage of 35.9× and dbSNP as filters to remove common variants. To identify candidate pathogenic mutations, we used SVA (http://www.svaproject.org/) to group all variants into functional categories. We expected that the variant responsible for MC would be rare and would not be found in dbSNP or in the control sequenced genomes. We also expected that it would be a severe functional variant, and thus concentrated on those variants that resulted in protein truncation: nonsense mutations and frameshifting indels.

## Supporting Information

Text S1 Supplementary methods and figures.
(3.28 MB DOC)
