# Supplementary material for: Whole-Genome Sequencing of a Single Proband Together with Linkage Analysis Identifies a Mendelian Disease Gene
Source: PLoS Genet. 2010 Jun 17;6(6):e1000991. doi: 10.1371/journal.pgen.1000991 (PMC2887469; doi:10.1371/journal.pgen.1000991)
Supplement: Text S1 — Supplementary methods and figures. (3.28 MB DOC) [file pgen.1000991.s001.doc]

Supporting Online Material:

Supplementary Methods and Figures

**METHODS**

**Study Subjects**

The study subjects included individuals from two families with metachondromatosis (MC). Subjects were identified and recruited from the Johns Hopkins Hospital Genetics Clinic. Family 1 is a four-generation family comprised of 12 individuals for whom we had DNA. Seven individuals were classified as "affected" and five were "unaffected”. Family 2 is a three-generation family of three "affected" individuals and seven "unaffecteds" for whom we had DNA.

**Additional Phenotypic Information**

MC is an autosomal dominant condition characterized by simultaneous exostoses (osteochondromas), commonly of the hands and feet and enchondromas of long bone metaphyses and iliac crests. Unlike the osteochondromas of hereditary multiple exostoses (MES) that typically point away from the adjacent epiphysis and rarely affect the hands or feet, those of MC point toward the epiphyses and usually present on the hands and feet [1,2]. Additionally, MC exostoses may regress or even resolve over time [1-3] while those in MES usually persist. Though palpable, the exostoses of MC may not be calcified and therefore may be radiolucent [4], in part depending on the timing of the clinical exam and radiography in the lifespan of a given lesion. The enchondromas of MC are similar to those of Ollier’s disease (also known as multiple enchondromatosis) but the latter disorder usually lacks exostoses. *EXT1* and *EXT2* mutations, located respectively at 8q24 and 11p11-p12, have been identified for MES in 70% of the cases [5]. Hopyan et al. [6] identified a mutant type I receptor for parathyroid hormone and parathyroid hormone-related protein (PTHR1) in enchondroma specimens from 2 of 6 individuals with Ollier enchondromatosis but Rozeman et al. [7] failed to find any mutation in the *PTHR1* and concluded that enchondromatosis is not caused by the *PTHR1* mutation found by Hopyan et al. [6].

**DNA Preparation**

We collected EDTA anticoagulated peripheral blood from the individuals in Families 1 and 2 and isolated DNA using the QIAGEN QIAamp Midi kit (Qiagen, Valencia, CA) according to the manufacturer’s instructions. We determined DNA concentration using a NanoDrop ND–1000 spectrophotometer and used it for the PCR, SNP genotyping and whole genome sequencing analyses described in this report.

**SNP Genotyping**

We genotyped seven individuals from Family 1 (IV-3, III-9, III-4, IV-2, V-1, III-8 and IV-9) on the Illumina HumanHap 550 Genotyping BeadChip v1.0 array at the SNP Center of The Johns Hopkins University Genetic Resources Facility. Samples were processed with the Infinium II assay protocol (Illumina, San Diego, CA) as previously described [8]. We genotyped the remaining five individuals from Family 1 (II-2, III-1, III-2, III-3 and IV-8) with the Illumina Human 610-Quad v1.0 Genotyping BeadChip at the Kennedy Krieger Institute Cytogenetics Laboratory according to the manufacturer's instruction for the Infinium HD Assay Super manual protocol. The Human 610-Quad BeadChip was imaged on the Illumina BeadArray Reader and data was processed with both Illumina GenomeStudio v2009.1 and KaryoStudio v.1.0.3 software modules. We analyzed the SNP data from the 12 genotyped individuals in Family 1 at a resolution of 50 Kb for CNVs (losses or gains) using the KaryoStudio v1.0.3 cnv partition v.2.4.3.0. and found none that were shared among all affected individuals.

**Linkage Analysis**

Linkage was run using a total of 10,763 informative SNPs at an average physical distance of 266 Kb. There were 3 inter-SNP intervals greater than 10 Mb (11, 24 and 32 Mb). In all cases, however, exclusion LOD scores were achieved (< -6), indicating adequate information for exclusion. There were 28 intervals smaller than 50 Kb, all with negative LOD scores, suggesting that there was no score inflation due to LD. Performing the analysis using the built in Merlin algorithm to correct for LD did not significantly change our results. Results obtained with different sets of SNPs under the same selection process described above from the genome wide array also did not significantly change our results. We were able to exclude linkage to 96% of the genome (LOD < -2) and 98.4% of the genome showed negative LOD scores. The remaining genome was under six distinct peaks on chromosomes 2 (3,895,999 - 10,054,387 bp), 5 (60,599,664 - 62,520,634 bp), 7 (39,477,148 - 43,124,493 bp), 8 (129,285,455 - 141,170,190 bp), 9 (111,064,384 - 119,264,831) and 12 (106,027,255 - 116,409,591 bp) (Figure 3). The maximum LOD scores at each peak were at 3 specific levels (1.07, 1.8 and 2.5) that correspond to the presence of 0, 1 or 2 non-penetrant individuals in the pedigree.

**SNP Duo**

We exported genotype data for each individual in Family 1 from GenomeStudio to the program SNPduo to visualize regions of shared alleles between two individuals using the principle of identity by state (IBS) [9]. We compared the affected individual (V-1) to all other genotyped members of the pedigree to observe genomic regions where 0, 1, or 2 alleles were shared between the two individuals. We identified one 7.7 Mb region on chromosome 12 from 107,213,140 bp to 114,871,593 bp for which each affected individual shared one allele and all but one unaffected individuals shared 0 alleles with V-1 in this region.

**Whole-genome Sequencing**

The DNA was prepared for sequencing according to the Illumina DNA sample preparation kit protocol. In brief, the DNA was randomly fragmented by nebulization followed by end repair, addition of a single A base, adaptor ligation, gel electrophoresis to isolate 400 bp fragments followed by PCR amplification. Next, the size-selected libraries were used for cluster generation on the flow cell. All prepared flow cells were run on the Illumina Genome Analyzer II using the paired-end module: for individual V-1 from MC pedigree 1, three flow cells produced paired-end reads that were each 75 bp long, one flow cell produced paired-end reads that were 75 bp for read 1 and 67 bp for read 2, one flow cell produced single reads of 75 bp each, and one flow cell produced single reads of 50 bp each. The average coverage for this individual was 31.8x, and the average coverage for the eight control genomes was 23.6x, 33.0x, 33.1x, 34.9x, 36.3x, 36.6x, 38.9x, and 50.5x.

DNA was aligned to the reference genome (NCBI Build 36 Ensembl release 50) using the BWA software (version 0.4.9) [10]. SAMtools (version 0.1.5c) was used to remove potential PCR duplicates via the rmdup (paired reads) and rmdupse (single reads) command [11]. It was also used for variant identification using the pileup command with the –c option and default settings. The variants were then filtered using SAMtool’s variation filter with the default settings but removing the filter for a maximum allowed coverage per variant by setting it to 10 million. All single nucleotide variants were screened for quality by only keeping those with a consensus score of at least 10. Indels were excluded if there were fewer than 3 reads supporting the non-reference allele.

We compared the SNPs called by whole-genome sequencing for individual V-1 from MC pedigree 1 to those called with the Illumina Human 610-quad beadchip; the sequenced individual was genotyped with this chip at the Duke Center for Human Genome Variation Genomic Analysis Facility for quality control purposes. We found that of the 300,026 SNPs with non-reference genotypes on the chip, 99.6% were found to have matching genotypes by whole-genome sequencing. Eighty-four percent of the mismatches were variants called as homozygous by whole-genome sequencing but heterozygous by the HumanHap610, which can happen when low coverage at a site results in only one allele being seen. Of the 280,274 SNPs with reference genotypes on the chip, 98.2% were found to have matching genotypes by whole-genome sequencing.

**Segregation of the *PTPN11* deletion in individuals from Family 1**

To analyze the segregation of c.514_524del11 in individuals from Family 1, we performed PCR using standard methods. The PCR condition was 35 cycles (initial denaturation at 94°C for 3 min followed by 35 cycles of 94°C for 30 sec, 56°C for 30 sec and 68°C for 1 min followed by a terminal extension at 68°C for 10 min). The reaction mixture (50 µl total volume) contained 1 µl of gDNA (150 ng/ µl), 5 µl of 10x AccuPrime PCR Buffer II, 1 µl of each primer at 10 µM, and 1 µl of AccuPrime Taq DNA Polymerase. The forward primer was 5’-GGAGAGCAATGACGGCAAGT-3’ (from Chr12 111375523 bp to 111375542 bp) and the reverse primer was 5’-GTAACATCTTGCCAGACCCAT-3’ (from Chr12 111375594 bp to 111375614 bp). The products of wild type *PTPN11* exon 4 (91 bp) versus c.514_524del11 (80 bp) were separated by electrophoresis in a NuSieve 2.5:1 agarose gel (Supplemental Figure 3 where individuals III-8, III-2, IV-9, and IV-2 have only the wild type 91 bp product and individuals II-2, III-9, III-4, III-3, III-1, IV-8, IV-3, and V-1 are heterozygotes with both the 91 bp wild type product and the mutant 80 bp product. We confirmed these results by direct Sanger sequencing of the amplified products in both directions.

To analyze the segregation of p.R138X in Family 2, we performed PCR amplification of *PTPN11* exon 4 plus ~ 50 bp of flanking intronic sequence using a forward primer corresponding to Chr12 111375321 bp to 111375343 bp (5’-TGAAAGAACAACATGAACCCATA-3’) and a reverse primer corresponding to Chr12 111375616 bp to 111375635 bp (5’-CAGCAGAAAAATCACCCAAA-3’) and the same PCR protocol as above. We restricted the PCR products with *Rsa*I using the conditions recommended by the supplier (New England Biolab). The p.R138X mutation alters an *Rsa*I recognition site: amplified products from the wild type allele are cut into two fragments of 139 and 176 bp; while products of the mutant allele are uncut (315 bp). Following digestion, we separated the products by electrophoresis in 1% agarose gels (Supplementary Figure 4). We also confirmed these results by directing Sanger sequencing of the PCR products in all individuals in both directions (Supplementary Figure 4).

**Sanger sequencing of candidate genes**

We sequenced directly in both directions all annotated exons plus flanking intronic splice acceptor and donor sites of *PTPN11* in amplified products of the genomic DNA from an affected individual (III-4) in family 2 using an ABI 3100 Genetic Analyzer, Applied Biosystems, CA) according to the company-recommended protocol.

**Sequencing of PTPN11 exon 4 in controls**

Exon 4, up to 5bp into the flanking introns, was successfully sequenced in 469 controls. The primers were designed using Primer3 (http://frodo.wi.mit.edu/primer3/) and were ordered from IDT (IA). The sequences were F1, 5’-ATGGACATCTCTCTGGGAAA-3’; R1, 5’- GACCCATTTTTCAACTGGAG-3’; F2, 5’-TTTCTGTCTCAGGTGGGATT-3’; R2, 5’- AAAGATTTGGGTCACCAGAC -3’. F2 and R2, flanking the exon, were used for PCR amplification. DNA was amplified using standard PCR techniques. Eighty-one controls underwent PCR amplification as follows: 95°C for 5 min; 20 cycles of 95°C for 30 sec, 60°C (decreasing by 0.5°C each cycle, finally reaching 50°C) for 30 sec, and 72°C for 30 sec; 30 cycles of 95°C for 30 sec, 55°C for 30 sec, and 72°C for 30 sec; and 72°C for 7 min. The other 388 samples underwent PCR amplification as follows: 95°C for 7 min; 37 cycles of 94°C for 30 sec, 55°C for 30 sec, and 72°C for 1 min; and 72°C for 7 min. All samples were then cleaned up using Sephadex G-100; the recovered product was subjected to a round of cyclo-sequencing by the ABI Big Dye V1.1 method (25 cycles of 96°C for 10 sec, 50°C for 5 sec, and 60°C for 4 min) and then cleaned up using Sephadex G-50. The sequencing primer used for the set of 81 samples was F2, while the set of 388 samples was sequenced using both F1 and R1 in separate reactions (these primers were too close to the ends of the exon for either of them to capture sequence for the entire exon alone). The products were then run on the ABI 3730 sequence analyzer (Applied Biosystems, CA) and the traces were examined using Sequencher (Gene Codes, MI).

1. Lachman RS, Cohen A, Hollister D, Rimoin DL (1974) Metachondromatosis. Birth Defects Orig Artic Ser 10: 171-178.

2. Bassett GS, Cowell HR (1985) Metachondromatosis: A report of four cases. J Bone Joint Surg Am 67: 811-814.

3. Koslowski K, Scougall JS (1975) Aust Paediatri J 11: 42-45.

4. Kennedy LA (1983) Metachondromatosis. Radiology 148: 117-118.

5. Bovee JVMG, Hameetman L, Kroon HM, Aigner T, Hogendoom PCW (2006) EXT-related pathways are not involved in the pathogenesis of dysplasia epipysealis hemimelica and metachondromatosis. J Pathol 209: 411-419.

6. Hopyan S, Gokgoz N, Poon R, Gensure RC, Yu C et al. (2002) A mutant PTH/PTHrP type I receptor in enchondromatosis. Nat Genet 30: 306-310.

7. Rozeman LB, Sangiorgi L, Briaire-de Brujn IH, Mainil-Varlet P, Bertoni F et al. (2004) Enchondromatosis (Ollier disease, Maffucci syndrome) is not caused by the PTHR1 mutation p.R105C. Hum Mutat 24: 466-473.

8. Miller ND, Nance MA, Wohler ES, Hoover-Fong JE, Lesi E et al. (2009) Molecular (SNP) analyses of overlapping hemizygous deletions of 10q25.3 to 10qter in four patients: Evidence for HMX2 and HMX3 as candidate genes in hearing and vestibular function. Am J Med Genet 149A: 669-680.

9. Roberson EDO, Pevsner J (2009) Visualization of shared genomic regions and meiotic recombination in high-density SNP data. PLoS One 4:e6711.doe:10.1371/journal.pone.0006711.

10. Li H, Durbin R (2009) Fast and accurate short read alignment with Burrows-Wheeler transform. Bioinformatics 25: 1754-1760.

11. Li H, Handsaker R, Wysoker A, Fennell T, Ruan J et al. (2009) The Sequence Alignment/Map format and SAMtools. Bioinformatics 25: 2078-2079.

**Supplementary Figure legends**

**Supplementary Figure 1:**

A. Antero-postero view of the pelvis of individual V-1 in Pedigree 1 at age 3 years showing irregular iliac crest bilaterally caused by enchondromas and enchondromas of the proximal femurs bilaterally.

B. Skin of dorsal thorax in individual I-2 in Pedigree 2 at age 71 years showing multiple lentigines.

**Supplementary Figure 2:**

Data from SNPduo for chromosome 12 (p13.33-q24.33; left to right, respectively). Proband V-1 compared to 9 other individuals from family 1. Regions of chromosome 12 with no shared material between the two individuals being compared are identity by state 0 (IBS0). These regions are displayed in red. Regions with one shared allele are IBS1 and are displayed in blue. The area highlighted in yellow is a 7.7 Mb region from 107,213,140-114,871,593 bp that is shared by all affected individuals and 1 individual (III-1) who had no evidence of MC as an adult. Individuals V-1 and IV-9 share one allele for the entire chromosome 12 because IV-9 is the mother of V-1.

**Supplementary Figure 3:**

Gel electrophoresis of PCR products of *PTPN11* exon 4 from members of Pedigree 1. The wildtype amplicon is 91 bp and the c.514_524del11 amplicon is 80 bp. The numbered boxes above each lane refer to Pedigree 1 (see Figure 2A). Affected individuals are indicated by the heavy border; unaffected individuals by the thin border.

**Supplementary Figure 4:**

Gel electrophoresis of PCR products of *PTPN11* exon 4 from members of Pedigree 2 following digestion with *Rsa*I. The wildtype allele is cut by *Rsa*I into two fragments 176 and 139 bps in length. The amplicon derived from the p.R138X allele is not cut by *Rsa*I and is 315 bp in length. The numbered boxes above each lane refer to Pedigree 2 (see Figure 2B). Affected individuals are indicated by the heavy border; unaffected individuals by the thin border.

**Supplementary Figure 5:**

A frame-shifting deletion of 11 base pairs (red arrow) causes metachondromatosis (OMIM 263750) in Pedigree 1. Displayed sections (from top to bottom) are: (1) an ideogram of chromosome 12; (2) genes located from 111.33Mbp to 111.44Mbp, including *PTPN11*, with blue rectangles denoting exons and green rectangles denoting introns; and (3) identified insertions/deletions in this region. Data were annotated and visualized using the SVA software (http://www.svaproject.org/).

**Supplementary Figure 1**

**
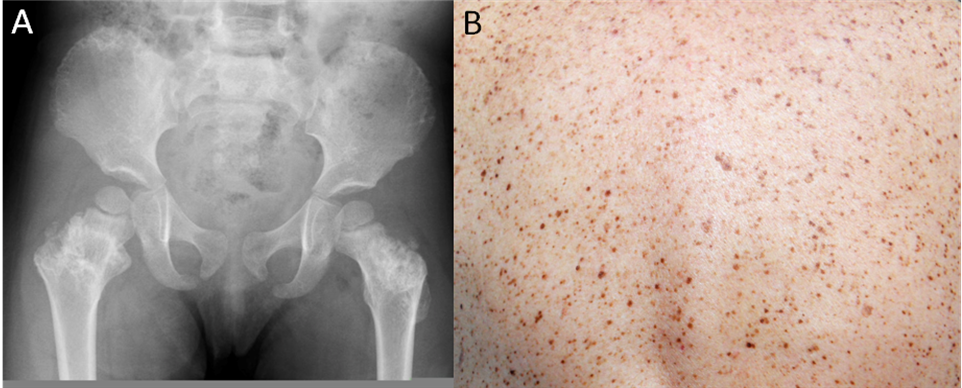
**

**Supplementary Figure 2**

**
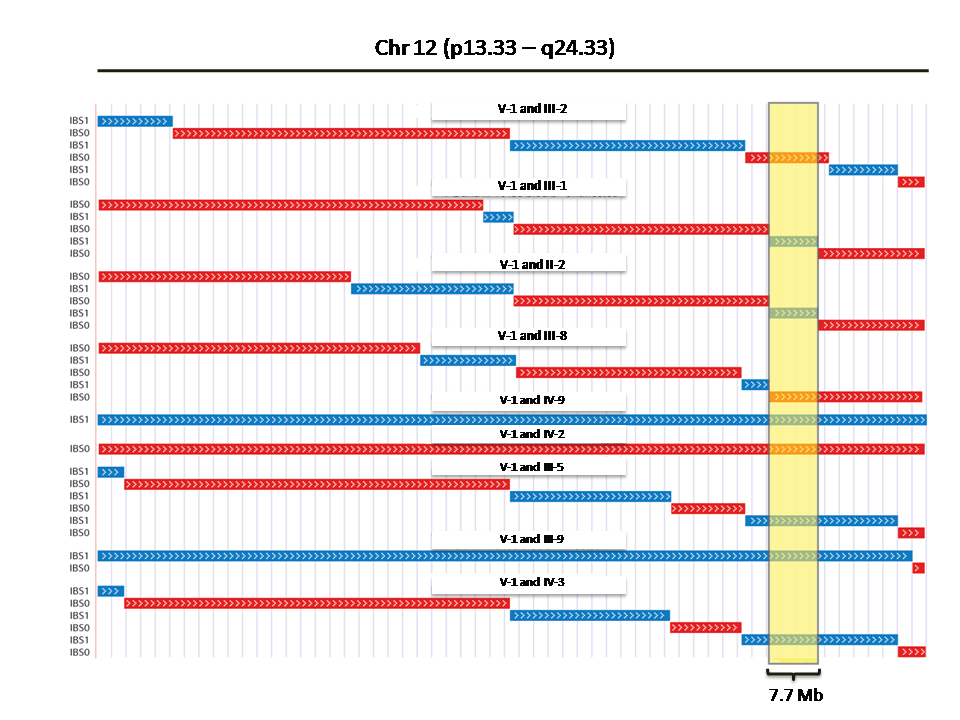
**

**Supplementary Figure 3**

**
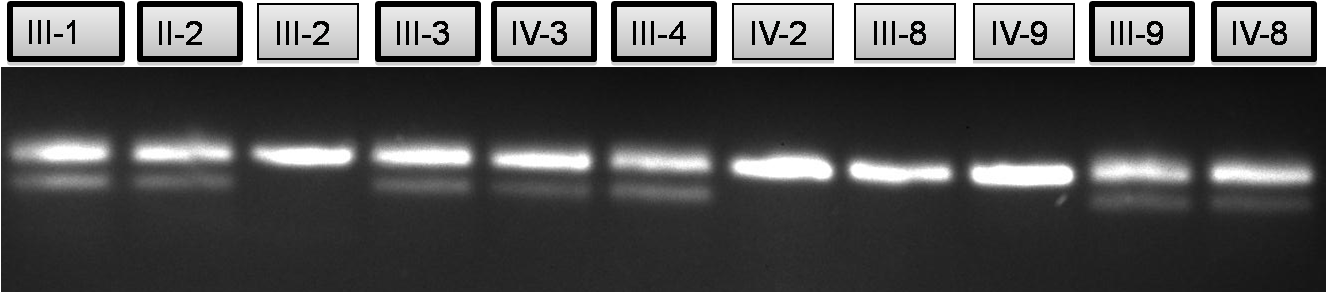
**

**Supplementary Figure 4**

**
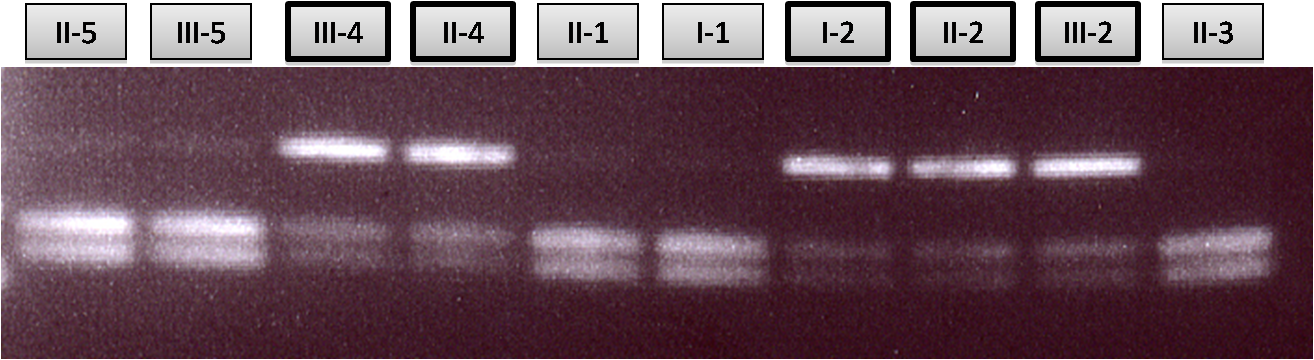
**

**Supplementary Figure 5**

**
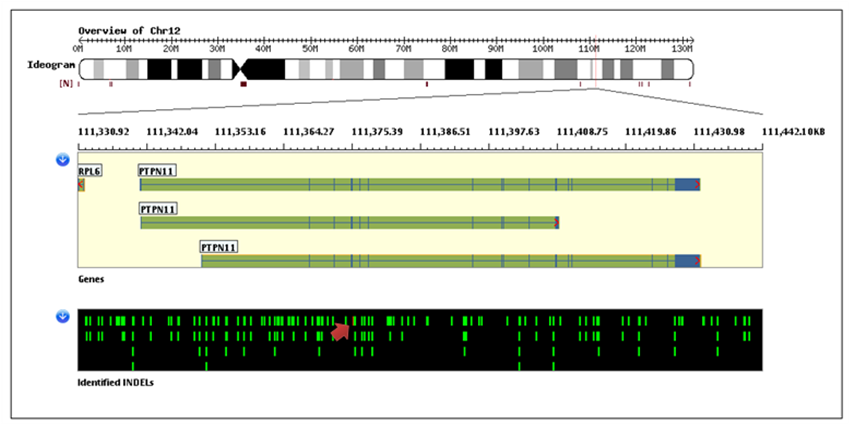
**
